# Preparation of RGD-modified liposomes encapsulated with shikonin and its targeted anti-melanoma effects

**DOI:** 10.3389/fonc.2025.1573628

**Published:** 2025-05-27

**Authors:** Hao Zhang, Ying Zhao, Tingting Chen, Xinliang Mao, Jiping Li, Li Fan, Min Li, Xianchun Wen

**Affiliations:** ^1^ Pharmacy School, Qiqihar Medical University, Qiqihar, Heilongjiang, China; ^2^ Medical Technology School, Qiqihar Medical University, Qiqihar, Heilongjiang, China; ^3^ Enrollment and Employment Department, Qiqihar Medical University, Qiqihar, Heilongjiang, China; ^4^ Public Health School, Qiqihar Medical University, Qiqihar, Heilongjiang, China; ^5^ Research Institute of Medicine and Pharmacy, Qiqihar Medical University, Qiqihar, Heilongjiang, China; ^6^ The Third Affiliated Hospital, Qiqihar Medical University, Qiqihar, Heilongjiang, China

**Keywords:** shikonin, melanoma, liposomes, RGD, α_V_β_3_ integrin

## Abstract

Melanoma is the most aggressive skin tumor, and conventional treatment is ineffective. Studies have shown that shikonin, derived from the traditional Chinese medicine *Lithospermum erythrorhizon*, has various anticancer activities. In this work, RGD- modified liposomes encapsulated with shikonin (RGD-Lip-SHK) were prepared by thin- film dispersion, which could highly recognize the integrin α_V_β_3_ on the surface of melanoma cells. RGD-Lip-SHK appeared as spheroid-like vesicles with a particle size of approximately 120 nm, and its ξ-potential was negative. RGD-Lip-SHK remained stable in serum within 48 h and possessed sustained-release effect. *In vitro*, compared with non -targeted liposomes (Lip-SHK), RGD-Lip-SHK was more efficiently taken up, had higher cytotoxicity, was better targeted to inhibit cell growth, migration, and invasion, and boost cell apoptosis by regulating the expression of Bcl-2 and Bax proteins in melanoma cells. *In vivo*, RGD-Lip-SHK had the strongest targeted anti-melanoma effect by α_V_β_3_-mediated endocytosis with a long circulation time and inhibited tumor growth in B16F10 tumor-bearing mice compared to other groups. Furthermore, the histology of major organs and the body weight of mice showed that RGD-Lip-SHK had less toxicity. In summary, these results indicated that RGD-Lip-SHK has great potential for the targeted treatment of melanoma, and is expected to become a novel and highly effective strategy for tumor-targeted therapy.

## Graphical Abstract

RGD-Lip-SHK actively delivers more drugs to melanoma cells via α_V_β_3_-mediated endocytosis and then induces apoptosis by regulating the expression of Bax and Bcl-2 proteins.

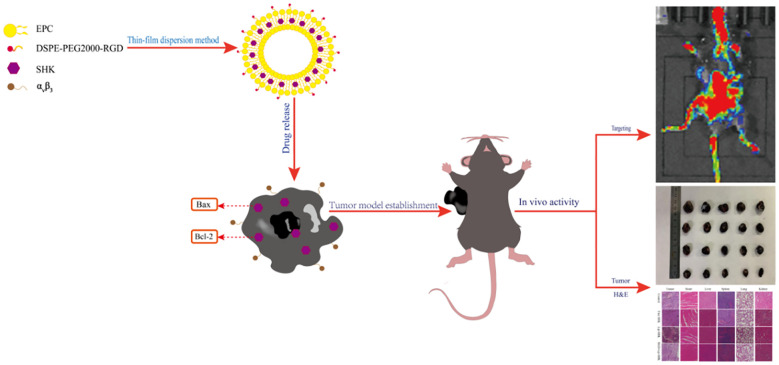

## 1 Introduction

Melanoma is the most lethal and malignant of all skin cancers, and the incidence is increasing year by year (1). According to the American Cancer Society’s evaluation, nearly 106, 110 cases were estimated in 2021 in the US. In China, despite the relatively low incidence of melanoma, it is increasing with a 3%-5% incidence per year (2). Currently, chemotherapy is the preferred treatment for primary melanoma, but its efficacy is greatly restricted due to dose limitations, side effects and less accumulation of tumour sites, thereby leading to poor prognosis. Therefore, it is very necessary to seek more efficient therapeutic drugs and strategies (3).

Shikonin, a bioactive ingredient derived from the roots of *Lithospermum erythrorhizon*, has been identified as a key component with potent pharmacological properties (4, 5). Studies have indicated that shikonin possesses many therapeutic effects, such as anti-inflammatory, antimicrobial, antiviral, cardiovascular protective, and antitumor (6–8). Particularly, the antitumour effect has attracted much attention in recent years. Past studies have shown shikonin possesses multiple anti-tumor effects as a promising anticancer agent, including lung cancer, gastric cancer, melanoma and colorectal cancer, etc (9–12). However, the high lipophilic property of shikonin leads to its defects such as poor water solubility, high toxicity, low bioavailability and short residence time, and these shortcomings limit its widespread application in the clinic (13, 14). Thus, the development of novel targeted drug delivery systems is expected to surmount these drawbacks.

Nanotechnology provides an optimal strategy to address the pharmacokinetic characteristics and side effects of the drugs like liposomes. Liposomes are near-spherical and hollow vesicles, which can serve as non-toxic and non-immunogenic natural carriers (15, 16). Since liposomes have many advantages such as low toxicity, enhanced permeability and retention (EPR) effect, biocompatibility, and the capacity to load lipophilic and hydrophilic agents, many investigators have focused on using liposomal carriers as an approach to reduce drug toxicity and/or target specific tumour cells (17–20).

Active targeting is the hotspot of nowaday research and has been shown to be a very effective anti-tumour strategy (21). To improve liposomes targeting, the PEGylated liposomes are further optimized by modifying the surface with ligands specific to cancer cells, thereby decreasing drug dosage and toxicity (22). Among them, the Arginine-glycine-aspartate (RGD), as a ligand on the surface of synthetic materials, is an efficiently and widely applied peptide sequence, the primary role of which can adjust cell adhesion through interaction with integrin receptors on the cell surface (23). As cell adhesion molecule, integrins play a vital role in regulating tumour cell growth, migration, and tumour angiogenesis, such as integrin α_V_β_3_ (24). Integrin α_V_β_3_ receptor appears overexpression on the surface of certain tumour cells, such as melanoma, hepatoma, breast cancer and glioblastoma (25). Because the RGD peptide is able to highly recognize and specifically bind to integrin α_V_β_3_, targeting the integrin α_V_β_3_ receptor to inhibit tumour growth and angiogenesis through its specific action will be a potential anticancer strategy (26, 27).

Based on the above, the aim of this study was to prepare RGD-modified liposomes encapsulated with shikonin (RGD-Lip-SHK), which targeted integrin α_V_β_3_ in melanoma cells, as shown in **Graphical Abstract**. The properties of RGD-Lip-SHK including the particle size, encapsulation efficiency, stability in serum and drug release were appraised. Moreover, cellular uptake of RGD-Lip-SHK was estimated in B16F10 melanoma and MCF-7 cells. In addition, antitumour effects of RGD-Lip-SHK were analyzed by tumour cell growth, migration and apoptosis assays *in vitro*. Meanwhile, a B16F10 tumour-bearing mice was built and used to appraise tumour growth inhibition of RGD-Lip-SHK. The biodistribution of RGD-Lip-SHK *in vivo* and histology of major organs, such as heart, lungs and liver in mice were also analysed. It is anticipated that the drug delivery system will be able to deliver more drugs to tumor sites through receptor-mediated endocytosis for tumor-targeted therapy.

## 2 Materials and methods

### 2.1 Materials

Shikonin (purity 99.8%) was sourced from Macklin Biochemical Technology Co., Ltd. (Shanghai, China). Egg phosphatidylcholine (EPC; purity 98%) was supplied from Aito Pharmaceutical Technology Co., Ltd. (Beijing, China). Cholesterol (Chol) was procured from Sigma-Aldrich Co., Ltd. (Shanghai, China). DSPE-PEG2000-RGD (purity > 97%) was synthesized by Shaanxi Zhongxiang Yunke Biotechnology Co., Ltd. (Shaanxi, China). Sephadex G-50 was supplied by Beijing Bairuide Biotechnology Co., Ltd. (Beijing, China). DMEM medium was provided by Zhongke Maichen Technology Co., Ltd. (Beijing, China). Coumarin-6 (Cou6) and 1,1′-dioctadecyl-3,3,3′,3′-tetramethylindotricarbocyanine iodide (DiR) were supplied from Beijing Bailingwei Technology Co., Ltd. (Beijing, China). Hoechst 33258 was obtained from Biosharp Biotechnology Co., Ltd. (Beijing, China). The Annexin V-FITC/PI apoptosis kit was supplied from Jiangsu Kaiji Biotech Co., Ltd. (Jiangsu, China). MTT was sourced from Saibai Ao Co., Ltd. (Beijing, China). Mouse polyclonal antibodies against human Bcl-2, Bax, and GAPDH were provided by Cell Signaling Technology, Inc. (Boston, MA, United States). A hematoxylin and eosin (H&E) staining kit was supplied from Shanghai Biyuntian Biotechnology Co., Ltd. (Shanghai, China).

### 2.2 Liposome preparation

Liposomes were prepared via the thin-film dispersion. A precisely measured EPC, Chol, DSPE-PEG2000, and SHK (weight: 30, 6, 6, and 0.5 mg, respectively) was dissolved in a round-bottomed bottle containing a mixture of dichloromethane and ethanol in a 1:3 volume ratio. This solution was then subjected to evaporation under pressure at 37°C for 1 h via spinning evaporator to generate lipid film. Subsequently, the film was mixed suspension with 2 ml of phosphate buffered saline (PBS) at 37°C and sonicated for 1 h to produce liposomal suspensions. Then the suspension was filtered through 220 nm polycarbonate membranes, then purified via gel filtration in a Sephadex G-50 column, finally nontargeted liposomes (Lip-SHK) was prepared.

A mixture of SHK, EPC, CHol, DSPE-PEG2000 and DSPE-PEG2000-RGD (weight: 30, 6, 3, 3, and 0.5 mg, respectively) was prepared into RGD-modified liposomes encapsulated with shikonin (RGD-Lip-SHK) using the same method. Meantime, blank liposomes, Cou6 and DiR liposomes was also produced as above.

### 2.3 Liposomes characterization

Liposomes characterization including particle sizes, PDI and ξ-potential was assayed by dynamic light scattering (DLS) (PSS, CA, USA). Liposomal suspension was dropped onto a copper mesh and stained with 2 % phosphotungstate acid, then liposomes morphology was observed using transmission electron microscopy (TEM) (Hitachi, Tokyo, Japan). Liposomes were placed in serum-containing PBS and stored in 4°C dark. The particle sizes of liposomes in serum were determined at different time points (0, 2, 4, 6, 8, 10, 12, 24, 36 and 48 h) as described above, and liposomes stability was examined. The encapsulation efficiency (EE %) of SHK was measured by high performance liquid chromatography (HPLC) (Waters, Massachusetts, USA). Briefly, liposomes were filtered and purified to remove free SHK as above method, then purified liposomes and unpurified liposomes were dissolved in methanol for demulsification. Samples were diluted with acetonitrile, centrifuged for 5 min and filtered, the supernatant was measured to determine the total weight of SHK in purified liposomes or unpurified liposomes using HPLC. The encapsulation efficiency (EE %) was computed using following Equation 1:


(1) $$\begin{array}{c} \text{EE}\%=\text{the}\;\text{total}\;\text{amount}\;\text{of}\;\text{SHK}\;\text{in}\;\text{purified}\;\text{liposomes}/\\ \text{the}\;\text{total}\;\text{amount}\;\text{of}\;\text{SHK}\;\text{in}\;\text{unpurified}\;\text{liposomes}\\ \times 100\%. \end{array}$$

### 2.4 *In vitro* SHK release

Drug release behavior was assessed via the dialysis method; 1 mL of Free SHK, Lip-SHK, and RGD-Lip-SHK solution was moved into dialysis bags (Molecular Weight Cut Off 12,000–14,000 Da). The dialysis bags were blebbed into 30 mL of medium containing PBS (pH 7.4) and 1% Tween 80 and shaken horizontally at 37°C. At specific time points (0, 2, 4, 6, 8, 12, 24, 30, 36, and 48 h), 1 mL dissolution medium was obtained, and the same amounts of dissolution medium were supplemented. The released SHK was quantified by HPLC. The cumulative release amount of SHK were calculated by means of the Equation 2:


(2) $$\text{Cumulative drug release}\;(\%)={\text{X}}_{t}/{\text{X}}_{0}\times 100\%.$$

### 2.5 Cell culture and animals

The melanoma (B16F10) cells and breast cancer (MCF-7) cells were purchased from Shanghai Hongshun Biotechnology Co., Ltd. (Shanghai, China). B16F10 and MCF-7 cells were incubated in a DMEM medium (5% CO_2_, 37°C) with 1% penicillin/streptomycin and 10% Fetal Bovine Serum (FBS).

The C57BL/6 mice (5–6 weeks old) were supplied by Heilongjiang Yuheng Veterinary Technology Service Co., Ltd. (Heilongjiang, China). The mice were maintained in a germ-free environment and had free access to water and food. According to the guidelines of the Animal Experimental Ethics Committee of Qiqihar Medical University, animal experiments were performed.

### 2.6 *In vitro* cell internalization

To estimate the cell internalization of different Cou6 formulations, the uptake efficiencies of Cou6 were analyzed by flow cytometry; 1 × 10^5^ B16F10 or MCF-7 cells/well were incubated in a 6-well plate for 24 h; then treated with serum-free DMEM medium containing Cou6, Lip-Cou6, and RGD-Lip-Cou6 (Cou6 concentration, 100 ng/mL); and continued to incubate for 1 h. The cells were rinsed with PBS three times and measured by flow cytometry (Becton Dickinson, New Jersey, USA) at 224 nm.

In addition, laser confocal microscopy (Zeiss, Jeantown, Germany) was equipped with a Plan-Apochromat 20×/0.8 M27 objective and applied to observe cell internalization. The cells were treated as described above, and harvested cells were rinsed, fixed, and stained with Hoechst 33258 fluorescent dye for 30 min. The cells were photographed for analysis.

### 2.7 *In vitro* cell viability

Cell viability was assayed using the MTT method; 5 × 10^3^ B16F10 or MCF-7 cells/well were cultured in a 96-well plate. After incubation for 24 h, the culture medium was removed, each well was rinsed with PBS, and then Free SHK, RGD-Lip-SHK, and Lip-SHK were added into each well at different concentrations of 1.0, 2.0, 4.0, 8.0, 16.0, and 32.0 µM/L. After incubation for 24 h, a culture medium containing MTT (100 μg/well) was placed into a 96-well plate. After incubation for 4 h, 100 μL of Dimethyl Sulfoxide (DMSO) replaced the culture medium containing MTT solution to dissolve purple formazan crystals. The absorbance was determined at 490 nm using an ELISA reader (Tecan, Diken, Austria), and the IC_50_ was calculated. The cell viability was computed using Equation 3. The influence of blank liposomes on cell viability was analyzed by the same method.


(3) $$\begin{array}{c} \text{Cell viability}(\%)\\ =(\text{O}{\text{D}}_{\mathrm{drugs}}-\text{O}{\text{D}}_{\mathrm{blank}})/(\text{O}{\text{D}}_{\mathrm{control}}-\text{O}{\text{D}}_{\mathrm{blank}}). \end{array}$$

### 2.8 Wound healing assay

*In vitro*, the migratory capacity of the cells was measured by wound healing assay; 4 × 10^5^ B16F10 or MCF-7 cells/well were incubated into a 6-well plate. Reference lines were marked on the back of plate. When the cells were fully grown and covered the plate, a wound was created using a germ-free pipette tip. The cell debris was washed with PBS, and Free SHK, RGD-Lip-SHK, and Lip-SHK diluted with a serum-free medium (SHK concentration, 2 µM/L) were added to each well. Meanwhile, a blank group without drugs was set up, and cell wound photographs at 0, 12, and 24 h were taken using an inverted microscope (Zeiss, 5× objective lens, Axio Observer Al).

### 2.9 *In vitro* inhibition effect of migration and invasion

B16F10 and MCF-7 cells migration capacity was assayed via transwell migration assay. 5 × 10^4^ cells were placed into upper chamber containing serum -free medium and the lower chamber was supplemented with medium containing 10% FBS. Subsequently, Free SHK, RGD-Lip-SHK, and Lip-SHK (SHK concentration, 2 µM/L) were used to treat cells in the upper chamber for 24 h, with culture medium as a control. Then, a wet cotton ball was used to remove redundant cells in the upper chambers, and cells that migrated into the lower chamber were fixed with paraformaldehyde for 30 min and stained with 0.5% crystal violet for 30 min. Migrated cells were analyzed using an inverted microscope. In addition, the cell invasion was performed through a transwell invasion assay. Briefly, the experimental procedures were performed as described above, except that the transfer membranes were precoated with 30 µg/well Matrigel. After the cells were photographed, the densities were assayed using an ELISA reader at 570 nm.

### 2.10 *In vitro* cell apoptosis

The effects of drugs on cell apoptosis were appraised using an Annexin V-FITC/PI kit; 4 × 10^5^ B16F10 or MCF-7 cells/well were cultured in a 6-well plate for 24 h. After 24 h, the cells were treated with a culture medium containing Free SHK, RGD-Lip-SHK, and Lip-SHK (SHK concentration, 2 µM/L) for 24 h, with the culture medium as the control. After rinsing with PBS, the harvested cells were stained using Annexin V-FITC and PI following the product instructions. Cell apoptosis was analyzed using a FACScan flow cytometer.

### 2.11 Western blotting assay

The levels of the apoptosis proteins were assayed via Western blotting assay. B16F10 cells were incubated with a culture medium containing RGD-Lip-SHK and Lip-SHK (SHK concentration, 2 µM/L) for 24 h. Then, the proteins were extracted using Sodium Dodecyl Sulfate-Polyacrylamide Gel Electrophoresis (SDS-PAGE) separation and transferred onto Polyvinylidene Fluoride (PVDF) membranes. The immunoblotted membranes were treated with mouse polyclonal antibodies against human Bcl-2 and Bax overnight and then cultured with secondary antibodies for 2 h, with GAPDH as a control. The protein bands were analyzed using the ChemiDoc™ MP imaging system (Bio-Rad, Hercules, CA, United States).

### 2.12 *In vivo* targeting imaging and biodistribution of liposomes

*In vivo* targeting effects of liposomes were estimated using an imaging system. Male C57BL/6 mice were subcutaneously inoculated with a B16F10 cell suspension (1 × 10^6^ cells). After tumors had grown to approximately 400 mm^3^, the experimental animals were randomly divided into the Free DiR group, RGD-Lip-DiR group, and Lip-DiR group (n = 3). The tumor-bearing mice were injected with different DiR formulations (100 μg DiR) via the tail vein. After anesthesia, *in vivo* distribution images of the mice were captured using an IVIS Lumina Series III *in vivo* imaging system (PerkinElmer, Waltham, MA, United States) at 0.5, 2, 6, 10, 24, 36, and 48 h. After the animals were euthanized, the tumors and the major organs were harvested for further study.

### 2.13 *In vivo* antitumor effect and safety evaluation

To further investigate the targeted anticancer effects of liposomes, the tumor-bearing mice were established as described above. When the tumor volume reached approximately 500 mm^3^, the volume of the tumor was calculated using Equation 4. Mice were randomly divided into the Free SHK group, RGD-Lip-SHK group, Lip-SHK group, and saline group (n = 5). The mice were administered with different SHK formulations (SHK dosage, 7.5 mg/kg) and saline via tail vein injection every 2 days for a total of six times. The body weights and tumor volume of tumor-bearing mice were measured six times. On the 16th day, the animals were sacrificed, and the tumors and major organs were obtained. The tumor weights were measured, and changes in major organs were examined by H&E staining.


(4) $$\text{V}(\text{m}{\text{m}}^{3})=1/2\times \text{Length}\times {\text{Width}}^{2}.$$

## 3 Results

### 3.1 Liposomes characterization

**Figure 1:**
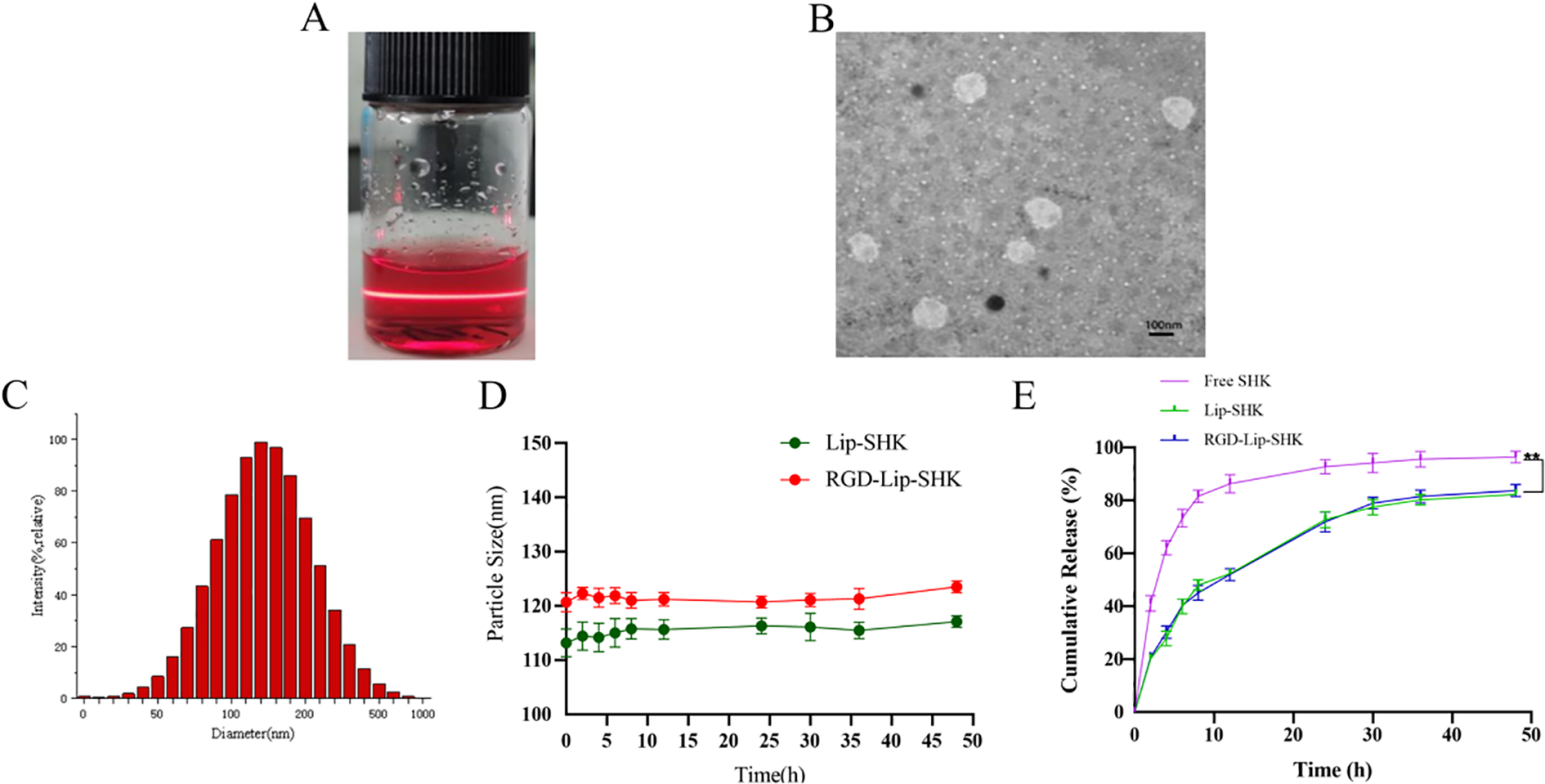
Characterization of liposomes. **(A)** Appearance form. **(B)** TEM micrograph. **(C)** Particle size distribution. **(D)** Change of particle size in serum for 48 h **(E)** *In vitro* release profiles of different SHK formulations at pH 7.4. Data are presented as mean ± SD (n = 3). ^**^ p < 0.01 compared with the Free SHK group. TEM, transmission electron microscopy.

The results indicated that Lip-SHK and RGD-Lip-SHK presented red transparent solution (**Figure 1A**) and appeared spheroid-like vesicles with particle size of 116.4 ± 2.36 and 121.2 ± 2.51 nm and homogeneous distribution with a polydispersity index (PDI) of 0.224 ± 0.03 and 0.216 ± 0.09, respectively (**Figures 1B, C**, **Table 1**). The encapsulation efficiencies of Lip-SHK and RGD-Lip-SHK were 92.4 ± 0.82 % and 93.6 ± 1.61 %, respectively (**Table 1**), suggesting that the RGD-modified liposomes possesses good drug loading capacity. In addition, the particle sizes of both liposomes within 48 h had no significant change, implying good stability in serum (**Figure 1D**).

**Table 1:** Characterization of liposomes (mean ± SD, n = 3).

Formulation	Particle size (nm)	PDI	ξ-Potential (mV)	EE %
Lip-SHK	116.4 ± 2.36	0.224 ± 0.03	−35.23 ± 4.17	92.4 ± 0.82
Lip-Cou6	116.1 ± 2.52	0.236 ± 0.01	−35.8 ± 0.64	95.42 ± 1.15
Lip-DiR	115.7 ± 1.89	0.241 ± 0.14	−35.09 ± 0.36	92.3 ± 1.48
RGD-Lip-SHK	121.2 ± 2.51	0.216 ± 0.09	−32.46 ± 3.15	93.6 ± 1.61
RGD-Lip-Cou6	120.7 ± 1.79	0.221 ± 0.08	−31.95 ± 0.23	96.26 ± 0.36
RGD-Lip-DiR	122.3 ± 1.15	0.217 ± 0.03	−31.87 ± 0.63	91.7 ± 0.45

PDI, polydispersity index; EE %, encapsulation efficiency.

As shown in **Figure 1E**, the drug release from Free SHK showed fast release, rapidly reaching more than 60% in the initial 5 h. However, SHK release from both liposomes displayed burst release, reaching approximately 30% in the first 5 h. Subsequently, the release behavior became slower and slower and went up to approximately 70% until 40 h, indicating that the SHK in both liposomes showed better stability and exhibited slow-release behavior. Meanwhile, Lip-SHK and RGD-Lip-SHK had a similar release behavior, suggesting that the RGD modifcation had no influence on drug release.

### 3.2 *In vitro* cell internalization

**Figure 2:**
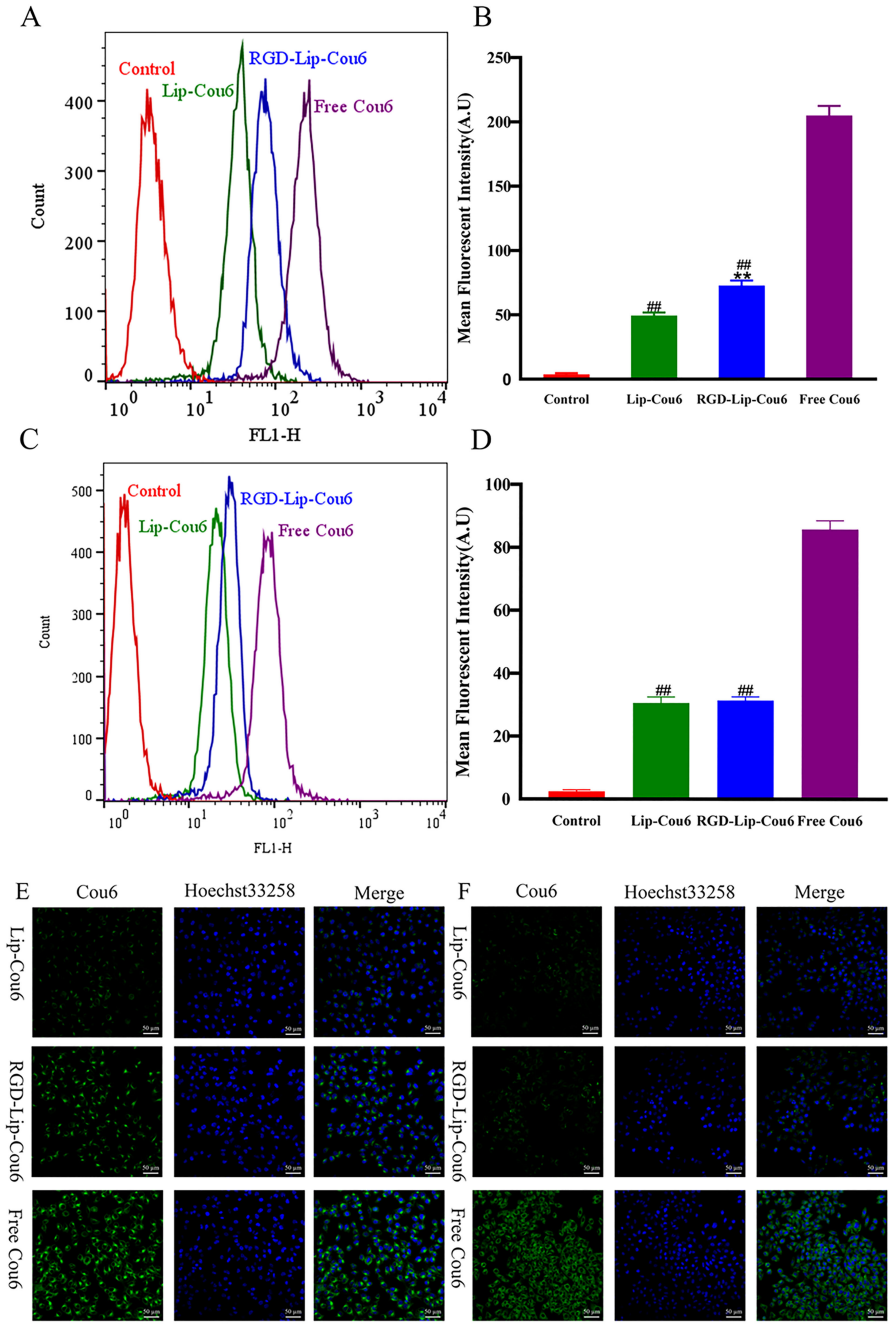
Cellular uptake of B16F10 **(A)** and MCF-7 **(C)** cells were assayed by flow cytometry. Mean fluorescence intensity of B16F10 **(B)** and MCF-7 **(D)** cells. Cellular uptake of B16F10 **(E)** and MCF-7 **(F)** cells was observed by laser confocal microscopy. The cell nucleus was stained with Hoechst 33258 (blue). Data are presented as mean ± SD (n = 3). ^##^p < 0.01 compared with the Free Cou6 group; ^**^p < 0.01 compared with the Lip-Cou6 group.

The cell internalization of different Cou6 formulations in the α_V_β_3_-overexpressed B16F10 and α_V_β_3_ low-expressed MCF-7 cells was evaluated via flow cytometry. After the cells were treated with Free Cou6, RGD-Lip-Cou6, and Lip-Cou6 for 2 h, the results demonstrated that Free Cou6 had the strongest fluorescence intensity in both cell lines (**Figure 2**). This result may be correlated with the sudden release of Free drug *in vitro*. Meanwhile, the fluorescence intensity of RGD-Lip-Cou6 was 1.64 times that of Lip-Cou6 in B16F10 cells (p < 0.01) (**Figure 2A**). In contrast, the fluorescence intensities of both liposomes had no distinct difference in MCF-7 cells (**Figure 2C**).

In addition, the laser confocal microscopy analysis displayed similar results, as shown in **Figures 2E, F**. The nuclei of B16F10 or MCF-7 cells and SHK were observed in the blue and green channels, respectively. The results revealed that the green fluorescence of B16F10 or MCF-7 cells treated with Free Cou6 was still the strongest. However, the green fluorescence of MCF-7 cells treated with Lip-Cou6 and RGD-Lip-Cou6 had no obvious difference. The results confirmed that RGD-Lip-Cou6 was well assimilated by B16F10 cells via α_V_β_3_-mediated internalization.

### 3.3 *In vitro* cell viability

**Figure 3:**
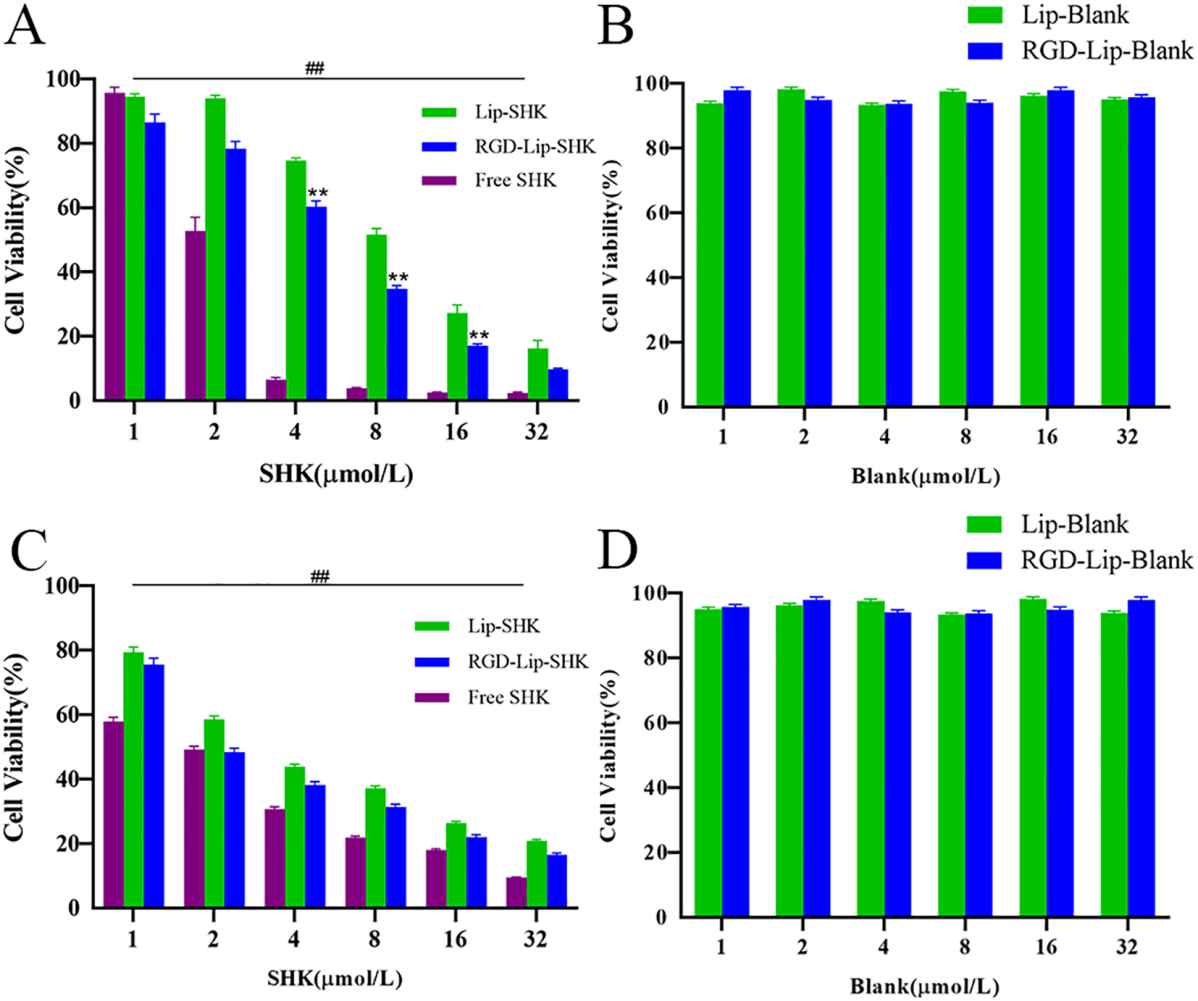
*In vitro* cell viability of B16F10 **(A)** and MCF-7 **(C)** cells after treatment of different SHK formulations. The cell viability of B16F10 **(B)** and MCF-7 **(D)** after treatment of blank liposomes. Data are presented as mean ± SD (n = 3). ^##^p < 0.01 compared with the Free SHK group; ^**^p < 0.01 compared with the Lip-SHK group.

The cell viabilities of blank liposomes (Lip-Blank), RGD-modified blank liposomes (RGD-Lip-Blank), Free SHK, RGD-Lip-SHK, and Lip-SHK were determined by MTT assay. As depicted in **Figures 3B, D**, Lip-Blank and RGD-Lip-Blank displayed no influence on the cell viability of both cells, with cell viability rates above 95%. Moreover, Free SHK, RGD-Lip-SHK, and Lip-SHK could all decrease cell viability in a concentration-dependent manner (**Figures 3A, C**). Among them, Free SHK had the strongest ability to inhibit B16F10 and MCF-7 cell growth with IC_50_ values of 2.19 ± 0.26 and 1.59 ± 0.10 µM, respectively (**Table 2**). In addition, the inhibition of RGD-Lip-SHK on B16F10 cells was stronger than that of Lip-SHK (IC_50_ value, 5.09 ± 0.36 and 8.94 ± 0.18 µM, respectively) (p < 0.01). However, no significant difference was observed in the inhibitory effect of both liposomes on MCF-7 cells (**Table 2**).

**Table 2:** Maximum half-inhibitory concentration (IC_50_) of different SHK formulations on B16F10 and MCF-7 cells.

Formulations	B16F10 IC_50_ (µM)	MCF-7 IC_50_ (µM)
Lip-SHK	8.94 ± 0.18^#^	2.73 ± 0.23^#^
RGD-Lip-SHK	5.09 ± 0.36^#^**	3.97 ± 0.27^#^
Free SHK	2.19 ± 0.26	1.59 ± 0.10

Data are presented as mean ± SD (n = 3).

^#^p < 0.05 compared with the Free SHK group.

^**^p < 0.01 compared with the Lip-SHK group.

### 3.4 *In vitro* inhibitory effect on migration and invasion

**Figure 4:**
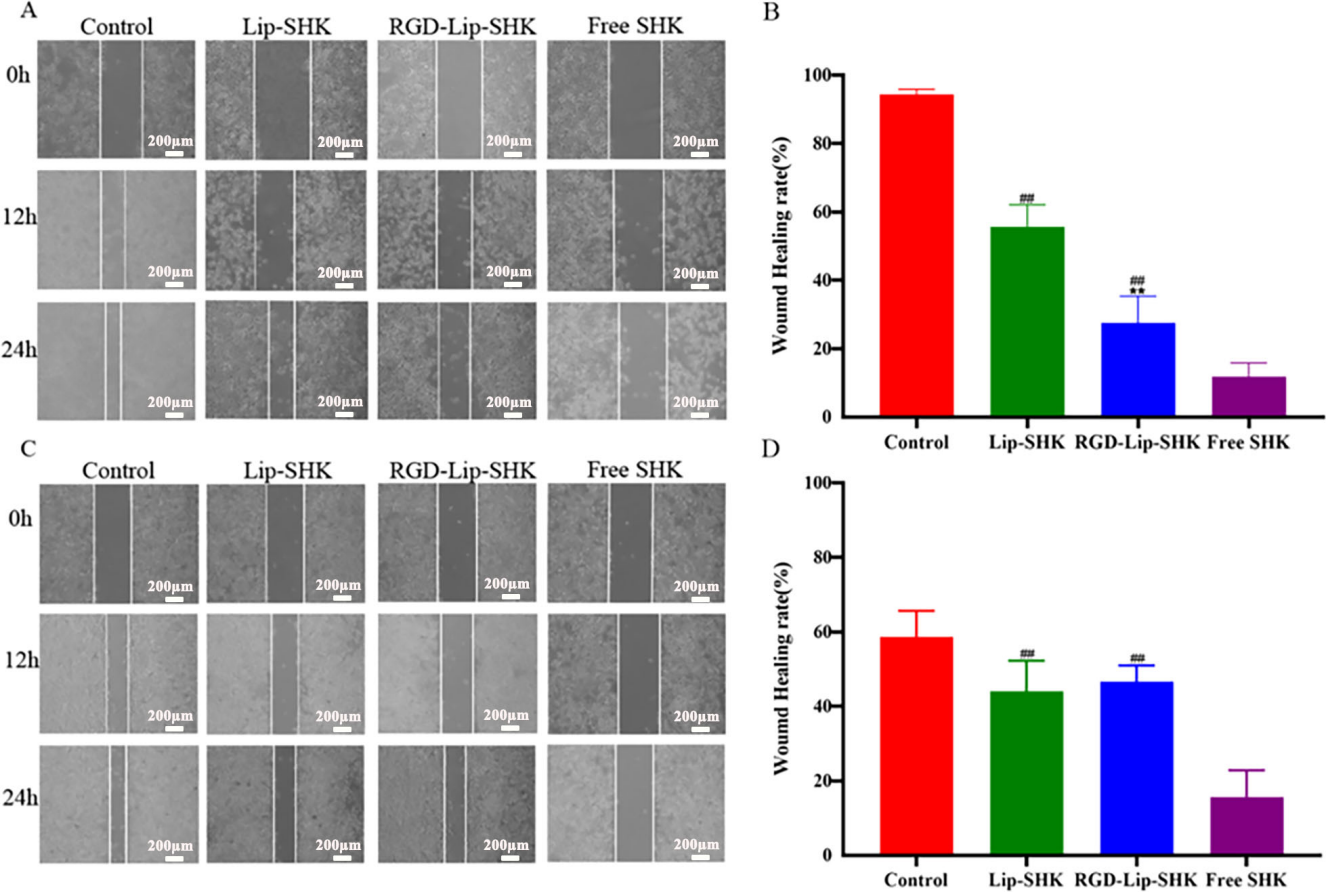
The effects of different SHK formulations on wound healing of B16F10 **(A)** and MCF-7 **(C)** cells. The wound healing rates of B16F10 **(B)** and MCF-7 **(D)** cells. Data are presented as mean ± SD (n = 3). ^##^p < 0.01 compared with the Free SHK group; ^**^p < 0.01 compared with the Lip-SHK group.

The effect of different SHK formulations on metastasis was analyzed by scratch wound healing and transwell migration assays. The results indicated that the wound healing rates of B16F10 and MCF-7 cells treated with Free SHK were the lowest and were 12.7% ± 1.42% and 15.69% ± 7.17%, respectively (**Figure 4**). Furthermore, the wound healing rate of B16F10 cells treated with RGD-Lip-SHK (32.25% ± 6.03%) was lower than that of cells treated with Lip-SHK (56.46% ± 4.78%) (**Figures 4A, B**), while that of MCF-7 cells treated with both liposomes had no distinct difference (**Figures 4C, D**).

**Figure 5:**
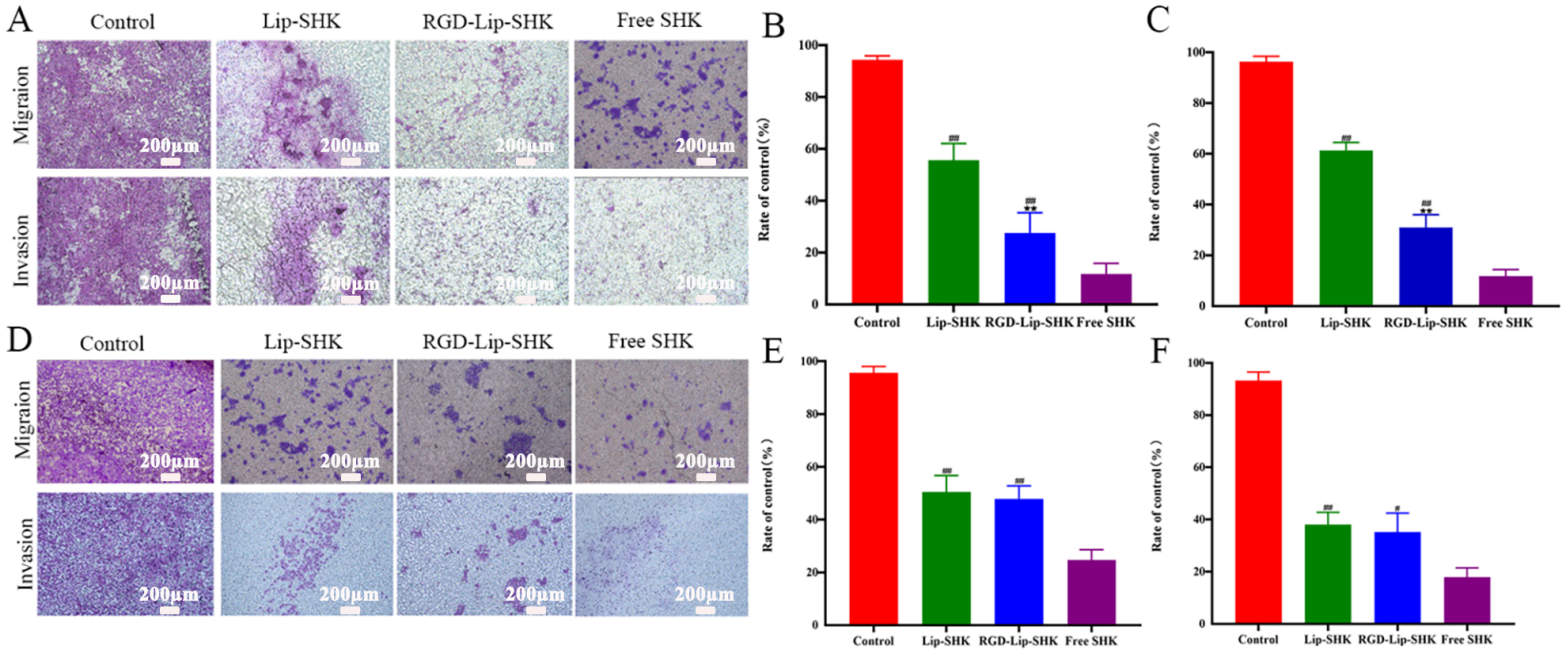
The effect of different SHK formulations on migration and invasion of B16F10 **(A)** and MCF-7 **(D)** cells. The relative migration cell in B16F10 **(B)** and MCF-7 **(E)** cells. The relative invasion cell in B16F10 **(C)** and MCF-7 **(F)** cells. Data are presented as mean ± SD (n = 3). ^##^p < 0.01 compared with the Free SHK groups; ^**^p < 0.01 compared with the Lip-SHK group.

Furthermore, the results of the transwell migration assay were similar to those of scratch wound healing (**Figures 5A, B, D, E**). In addition, the results indicated that Free SHK showed the strongest invasion inhibition ability for both cells (**Figures 5A, C, D, F**), and RGD-Lip-SHK had a higher ability to inhibit B16F10 cell invasion than Lip-SHK (p < 0.05) (**Figures 5A, C**). Meanwhile, no obvious difference was observed in the invasion inhibition of both liposomes on MCF-7 cells (**Figures 5D, F**).

### 3.5 *In vitro* cell apoptosis

**Figure 6:**
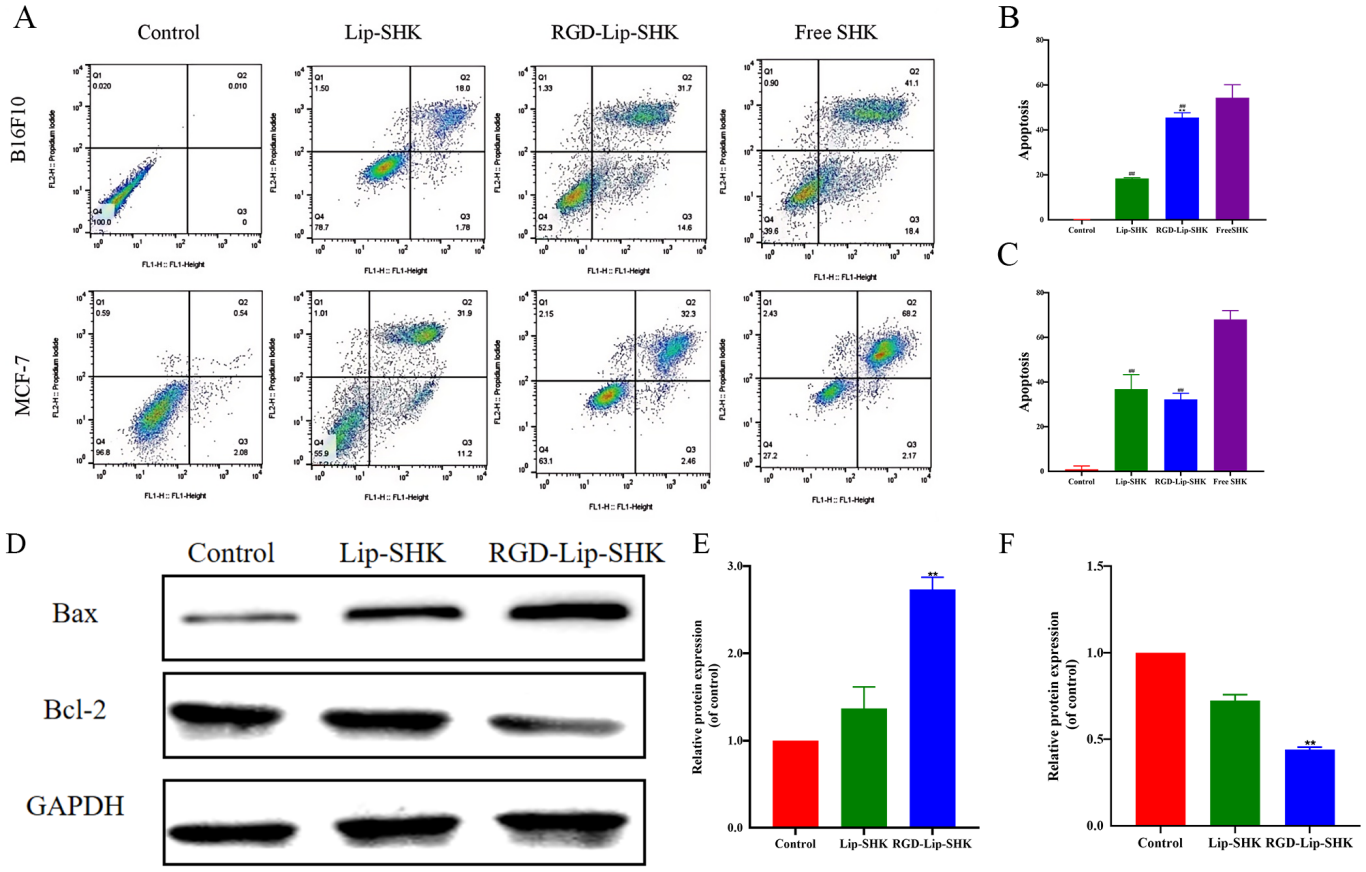
Apoptosis assay *in vitro*. **(A)** The apoptosis of B16F10 and MCF-7 cells after treatment with different SHK formulations. The apoptotic rates of B16F10 **(B)** and MCF-7 **(C)** cells. **(D)** The expression levels of Bax and Bcl-2 proteins in B16F10 cells. The analysis of Bax **(E)** and Bcl-2 **(F)** by Western blotting. Data are presented as mean ± SD (n = 3). ^##^p < 0.01 compared with the Free SHK group; ^**^p < 0.01 compared with the Lip-SHK group.

The effect of different SHK formulations on cell apoptosis was estimated by flow cytometry. The results revealed that Lip-SHK, RGD-Lip-SHK, and Free SHK could all significantly induce apoptosis in both tumor cells compared to the control group. Among them, the apoptotic rates of B16F10 and MCF-7 cells treated with Free SHK were the highest at 54.3% ± 5.78% and 68.05% ± 3.94%, respectively (**Figure 6A**). In addition, the apoptotic rate of B16F10 cells treated with RGD-Lip-SHK (45.5% ± 2.11%) was higher than that of cells treated with Lip-SHK (18.4% ± 0.41%) (**Figures 6A, B**). Meanwhile, the effects of both liposomes on MCF-7 cells had no evident difference (**Figures 6A, C**). These results showed that RGD-modified liposome-loaded SHK could better promote B16F10 cell apoptosis through the interaction of RGD and α_V_β_3_ receptors, which were consistent with the results of cell viability and inhibition of migration and invasion as described above, suggesting the relevance between intracellular SHK level and tumor inhibition.

The results of Western blotting assayshowed Lip-SHK and RGD-Lip-SHK could significantly downregulate the expression of Bcl-2 protein, and obviously upregulate the expression of Bax protein compared to control group (**Figures 6D, E, F**). Meanwhile, the targeted regulation of RGD-Lip-SHK on apoptosis-related proteins was significantly stronger than that of Lip-SHK (*p* < 0.05). The results indicated that RGD-Lip-SHK had a strong target to promote apoptosis compared to Lip-SHK, suggesting the activation of mitochondrial apoptosis pathway might be a potential mechanism for anticancer effect of SHK.

### 3.6 *In vivo* targeted imaging and biodistribution of liposomes

**Figure 7:**
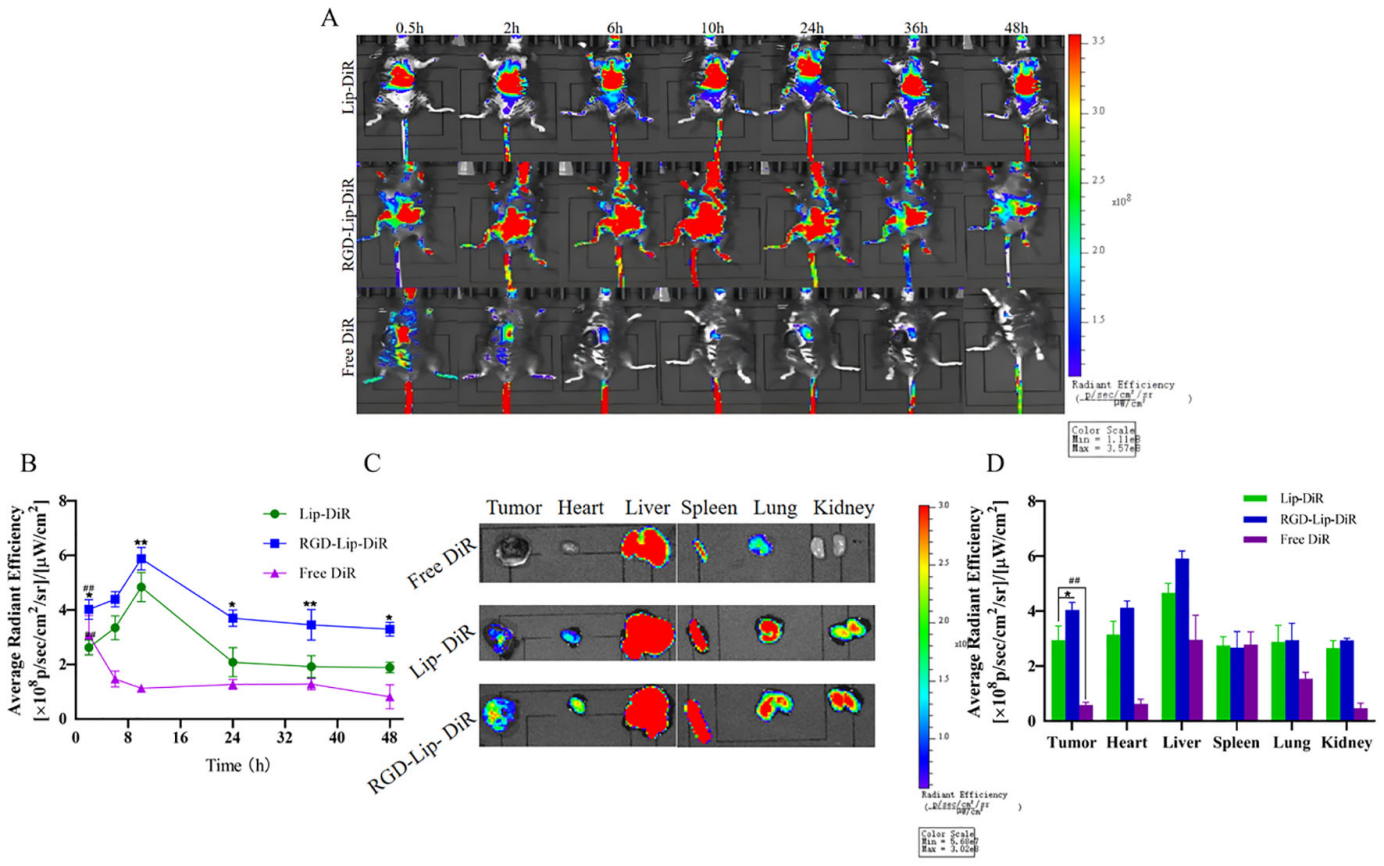
*In vivo* live imaging in C57BL/6 melanoma model. **(A)** *In vivo* fluorescence distribution imaging at different intervals after treatment with different DiR formulations. **(B)** The *in vivo* fluorescence intensity of Lip-DiR, RGD-Lip-DiR, and Free DiR at various time points. **(C)** The *ex vivo* fluorescence imaging of tumors and organs at 48 h. **(D)** The quantification of *ex vivo* fluorescence intensity in tumors and major organs. Data are presented as mean ± SD (n = 3). ^##^ p < 0.01 compared with the Free SHK group; ^*^p < 0.05 compared with the Lip-SHK group; ^**^p < 0.01 compared with the Lip-SHK group.

The C57BL/6 tumor-bearing mice were injected with Lip-DiR, RGD-Lip-DiR, and Free DiR through the tail vein and imaged at predetermined time intervals using an *in vivo* imaging system. As depicted in **Figures 7A, B**, after DiR injection for 0.5 h, DiR was distributed into the body via blood circulation in all groups, but the Free group accumulated very little DiR at the tumor site. In contrast, the accumulation of DiR at the tumor site in the Lip-DiR and RGD-Lip-DiR groups gradually increased from the second hour, was the strongest at 10 h, and began to weaken until after the 24th hour, while the fluorescence intensity in the RGD-Lip-DiR group was always the strongest.

Moreover, as shown in **Figures 7C, D**, the *ex vivo* images of tumor tissues showed that the fluorescence intensity of the RGD-Lip-DiR group appeared the strongest, followed by that of the Lip-DiR group, while the fluorescence signal of the Free DiR group was barely visible in the tumor site at 48 h, suggesting that RGD-Lip-DiR had stronger tumor-targeting properties due to the RGD specifically recognizing and binding α_V_β_3_ receptors (**Figures 7C,D**). Meanwhile, the fluorescence intensities of the Lip-DiR and RGD-Lip-DiR groups in major organs (liver, spleen, lung, kidney, and heart) were very strong, while those of the Free DiR group in major organs (liver, spleen, and lung) appeared weak at 48 h. These results showed that RGD-Lip-DiR possessed a long retention time, excellent biocompatibility, and targeting of mass accumulation at the tumor sites due to the modification of the RGD on the liposomal surface.

### 3.7 *In vivo* antitumor effect and safety evaluation

**Figure 8:**
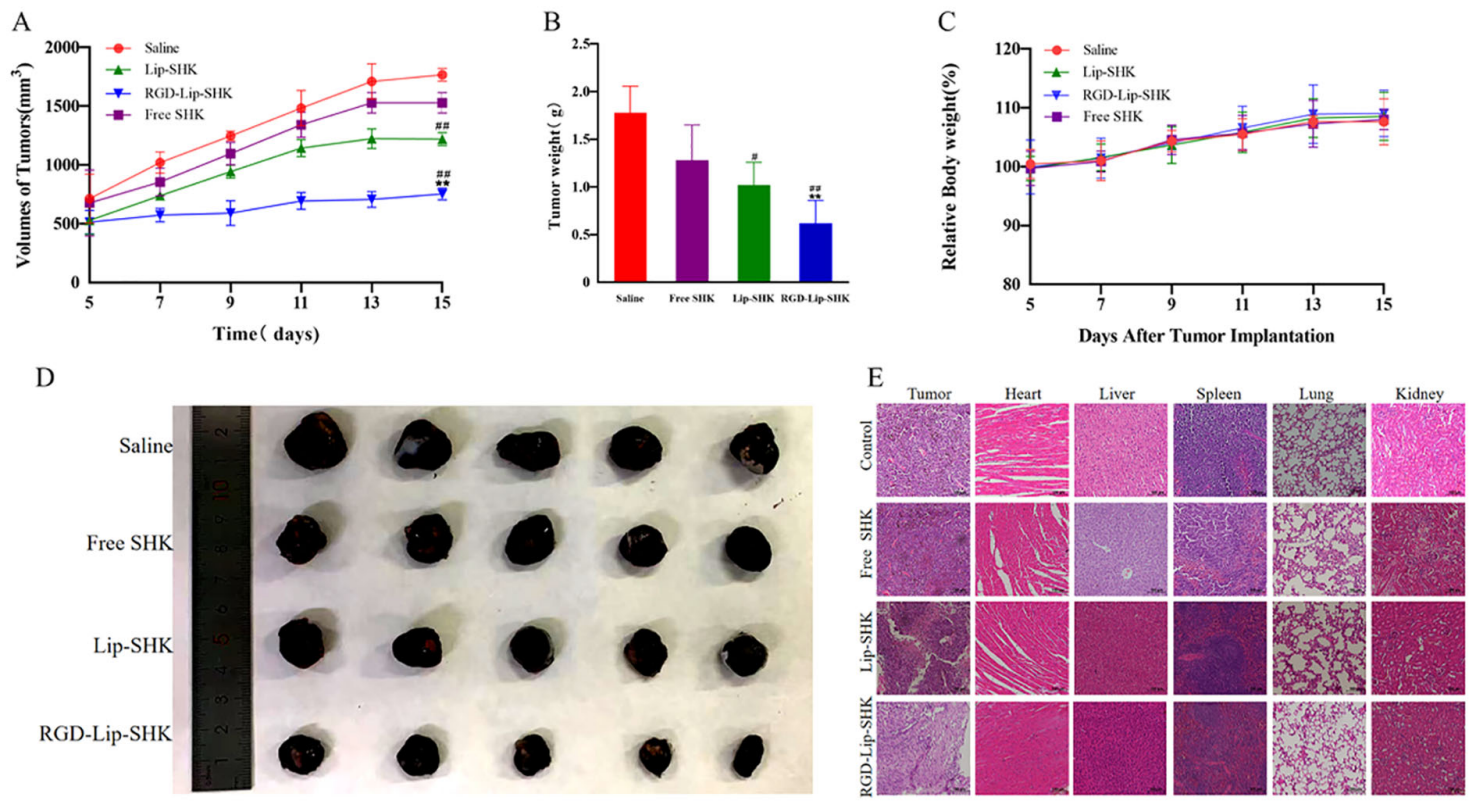
*In vivo* antitumor experiments. **(A)** The tumor growth curves. **(B)** The tumor weight change curves. **(C)** The body weight change curves during the experimental period. **(D)** Photographs of representative tumors on day 16. **(E)** H&E analysis of major organ tissues. Data are presented as mean ± SD (n = 5). ^#^p < 0.05 compared with the Free SHK group; ^##^p < 0.01 compared with the Free SHK group; **p < 0.01 compared with the Lip-SHK group. H&E, hematoxylin and eosin staining.

The antitumor efficacies of different SHK formulations were evaluated in C57BL/6 tumor-bearing mice. The tumor volume in the saline group grew rapidly to approximately 1,000 mm^3^ on the 7th day (in **Figure 8A**). Furthermore, the tumor growth curve of the RGD-Lip-SHK group had the most conspicuous inhibition of tumor volume, which was mainly attributed to the liposomal EPR effect and the targeting ability of the liposomes modified by RGD, while Free drugs and Lip-SHK also inhibited tumor growth to different degrees.

After the termination of the experiment, the isolated tumors were collected, weighed, and photographed. The results indicated that RGD-Lip-SHK had the strongest tumor-targeting inhibition compared to Free SHK and Lip-SHK (p < 0.01), and their tumor inhibition rates were 51.56% ± 0.24%, 18.10% ± 0.72%, and 32.70% ± 0.57%, respectively (**Figures 8B, D**). Thus, the RGD modification could enhance the permeability and accumulation of RGD-Lip-SHK at the tumor sites, thereby presenting the strongest targeted antitumor efficacy.

The body weight of the mice was measured every day to evaluate the side effects of different SHK formulations. The body weight of all mice showed no significant change, implying that SHK had no obvious systemic toxicity (**Figure 8C**). Simultaneously, the effect of different SHK formulations on tumors and major organs was appraised by H&E staining. In comparison to the saline group, the Free SHK, Lip-SHK, and RGD-Lip-SHK groups all induced extensive tumor cell degeneration and necrosis, but the effect of the RGD-Lip-SHK group was stronger. The pathological tissue examination of major organs showed that no distinct toxicity was observed in each group, indicating that the drug was safe (**Figure 8E**).

## 4 Discussion

As we all know, the physicochemical characterization of drug-loaded liposomes can affect their uptake, stability and metabolism (28). In this work, all liposomes were constructed via the thin-film dispersion (29). Liposomes had a average particle size of around 120 nm with PDI about 0.21, which could efficiently deliver SHK to the melanoma site via EPR effect. Liposomes exhibited a negative charge, which could ensures electrostatic repulsion among liposomes so that enhance their stability and prevent their aggregation or adhesion to vascular endothelium (30). Furthermore, more than 60 % of SHK was released from Free SHK group in initial 5 h and about 30 % of SHK was released from Lip-SHK and RGD-Lip-SHK. Subsequently, the release of SHK from both liposomes got slower and slower, and went up to about 70 % until 40 h, indicating that the SHK in both liposomes showed good stability and slow-release behavior. Sustained release is an important feature of liposomes, which can decrease the frequency of administration, increase the local concentration of drugs, and enhance drug efficacy (31).

The results of cell internalization indicated that Free cou6 had the strongest Fluorescent intensity in B16F10 and MCF-7 cells, implying that it directly crossed the cell membrane into the tumour cells, resulting in maximal intracellular accumulation over a short time period (32). The Fluorescent intensity of RGD-Lip-Cou6 was stronger than that of Lip-Cou6 in B16F10 cells, suggesting that a targeting effect of RGD peptide, which interacted with integrin αVβ3 to increase the cell internalization of the cou6 in the liposomes via αVβ3-mediated endocytosis (33, 34). It is well known that liposomes have many favorable properties, such as reducing the toxicity and side effects of Free drugs, improving drug bioavailability, prolonging circulation time, and enhancing targeted therapy (35). Cell viability experiments revealed that Lip-blank and RGD-Lip-blank were not toxic to B16F10 and MCF-7 cells, hinting lipid materials and RGD was safe and non-toxic (36). Meanwhile, Free SHK had the strongest cytotoxicity in both cells, while the cytotoxicity of RGD-Lip-SHK on B16F10 cells was stronger than that of Lip-SHK. Due to the single living environment of tumour cells in vitro, Free drugs entered cells directly and caused the greatest cytotoxicity via fast release, while RGD-Lip-SHK could actively target αVβ3 receptors overexpressed on B16F10 cell surface, and gradually deliver more SHK to tumour cells through slow-release behavior, further increasing the anticancer efficacy (37, 38). Migration and invasion are prominent pathological processes of tumour progression. In metastasis, tumour cells depart from their original location, cross basement membrane into the blood or lymph, and ultimately colonize at a distance (39). Our results demonstrated that Free SHK could well inhibit B16F10 and MCF-7 cells migration and invasion compared to RGD-Lip-SHK and Lip-SHK, while the migration and invasion inhibition of RGD-Lip-SHK on B16F10 cell were stronger than that of Lip-SHK, indicating RGD-Lip-SHK could target integrin αVβ3 on the surface of B16F10 cells and better exert the anticancer-targeted efficacy. Apoptosis is the death of cells under physiological or pathological conditions, induction of apoptosis in tumor cells is considered a common strategy for cancer treatment (40). In this study, the results revealed that Lip-SHK, RGD-Lip-SHK and Free SHK could all significantly induce apoptosis in both tumour cells compared to control group. Among them, the apoptotic rate of B16F10 and MCF-7 cells treated with Free SHK was the strongest. In addition, the apoptotic rate of B16F10 cells treated with RGD-Lip-SHK was higher than that treated with Lip-SHK. To further confirm whether RGD-Lip-SHK-induced apoptosis was mediated through the mitochondrial pathway, the apoptosis-related protein were analysed using Western blotting assay. Since Bcl-2 is a critical signaling pathway associated with mitochondrial apoptosis, the inhibition of Bcl-2 plays a important role in the antitumour (41, 42). The results indicated Lip-SHK and RGD-Lip-SHK could significantly downregulate the expression of Bcl-2 protein, and obviously upregulate the expression of Bax protein compared to control group in B16F10 cells. However, the targeted regulation of RGD-Lip-SHK on apoptosis-related proteins was significantly stronger than that of Lip-SHK, suggesting the activation of mitochondrial apoptosis pathway might be a potential mechanism for anti-melanoma effects.

Long residence time of the liposomes can keep from their degradation in vivo, reduce their toxicity, improve the pharmacokinetics and enhance antitumour efficacy (43). Based on the previous research (44), we found the peak concentration, area under plasma concentration-time curves, half-life, and mean residence time of RGD-Lip-SHK distinctly increased compared to those of free SHK in rats. In the study, after the C57BL6 tumour-bearing mice were injected with different DiR formulations through the tail vein, in vivo targeted imaging showed that the RGD-Lip-DiR group had the highest fluorescence density at the tumor site, followed by Lip-DiR, but Free group accumulated very little DiR at the tumour site. The ex vivo images of tumour tissues showed the fluorescence intensity of RGD-Lip-DiR group still appeared the strongest, while the fluorescence intensityof Free DiR group was barely visible in tumour site at 48 h. These results suggested that RGD-Lip-DiR possessed long residence time, excellent biocompatibility, and targeting of mass accumulation at the tumour sites due to the modification of the RGD on the liposomal surface (45).

Furthermore, the results of in vivo anti-melanoma experiments showed that RGD-Lip-SHK had the strong tumour-targeted inhibition compared to Free SHK and Lip-SHK. Interestingly, we found that in vivo anti-tumour results were somewhat inconsistent with in vitro. In vivo, the anti-tumour efficacy of RGD-Lip-SHK was significantly stronger than that of Free SHK and Lip-SHK, which may be mainly due to the scattered growth of tumour cells in vitro, while in vivo tumour cells grow in clusters. In addition, the tumour entities in vivo also show resistance to drugs because of the three-dimensional arrangement of tumour cell populations and the interaction of specific cells (46, 47). Moreover, the extracellular matrix secreted by tumour cells also hinders drug delivery to the tumour sites (48). Because the overexpression of integrin αVβ3 in melanoma cells, RGD-Lip-SHK could delivery more drugs to the tumour site through binding of the RGD and αVβ3 receptors. Meantime, RGD belongs to penetrating peptide homes, which can promote the penetration of RGD-Lip-SHK into the tumour interior (49, 50). The systemic toxicity of drugs has been a chief drawback that limits their clinical use (51). The changes in body weight are also an important marker of drug safety (52).The results revealed that the body weight of mice and the pathological tissue examination of major organs had no distinct change, suggesting RGD-Lip-SHK was safety. Therefor, RGD-Lip-SHK may be a potential candidate for anti-melanoma-targeted therapy, and it is worthy of further research on its clinical applicability.

## 5 Conclusion

In this study, we successfully prepared RGD-modified liposome encapsulated with shikonin (RGD-Lip-SHK), which had optimal particle sizes, high encapsulation efficiency, enhanced colloidal stability, and sustained release properties. *In vitro*, RGD-Lip-SHK could obviously inhibit multiplication, migration and invasion, and promote apoptosis by downregulating the levels of Bcl-2 and upregulating the levels of Bax in melanoma cells. In vivo biodistribution studies showed that the accumulation of liposomes in tumors was sustained for up to 48 hours compared to free DiR. The RGD-Lip-SHK achieved active tumor targeting through α_v_β_3_ recognition coupled with enhanced retention effects. *In vivo*, compared with Free SHK and Lip-SHK, RGD-Lip-SHK could effectively mediate active targeting, and accumulate substantially at the tumor site with long circulation, thereby presenting the strongest antitumour-targeted efficacy. Moreover, no distinct toxicity was observed in all C57BL6 tumour-bearing mice by histology of major organs and the weight of mice. This nanoplatform establishes a robust foundation for melanoma-targeted therapy through integrin α_v_β_3_ receptor-mediated endocytosis. In the future, we will further explore the mechanism of action of this nano- drug delivery system against melanoma, providing experimental and theoretical basis for the next clinical study.

## Data Availability

The original contributions presented in the study are included in the article/**Supplementary Material**. Further inquiries can be directed to the corresponding author.
