# Supplementary figures and images for: Preparation of RGD-modified liposomes encapsulated with shikonin and its targeted anti-melanoma effects
Source: Front Oncol. 2025 May 27;15:1573628. doi: 10.3389/fonc.2025.1573628 (PMC12148886; doi:10.3389/fonc.2025.1573628)

BAX 21KDa

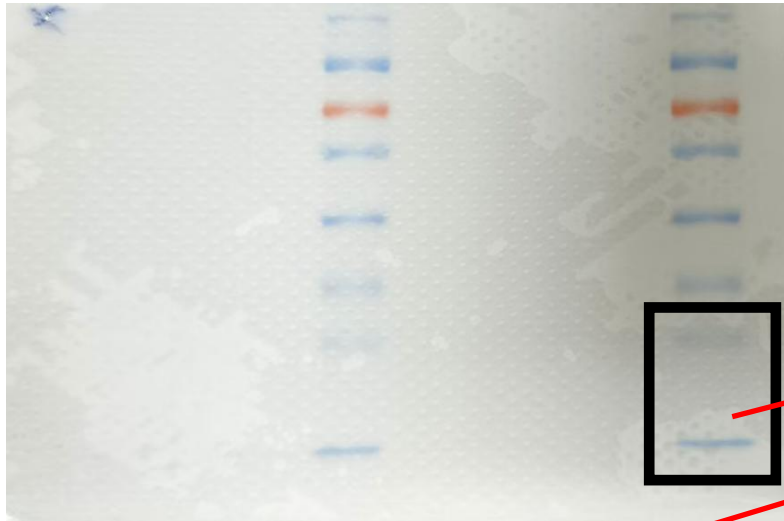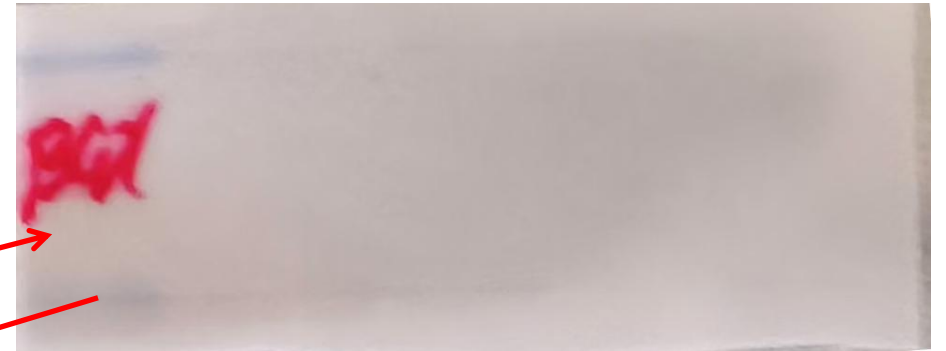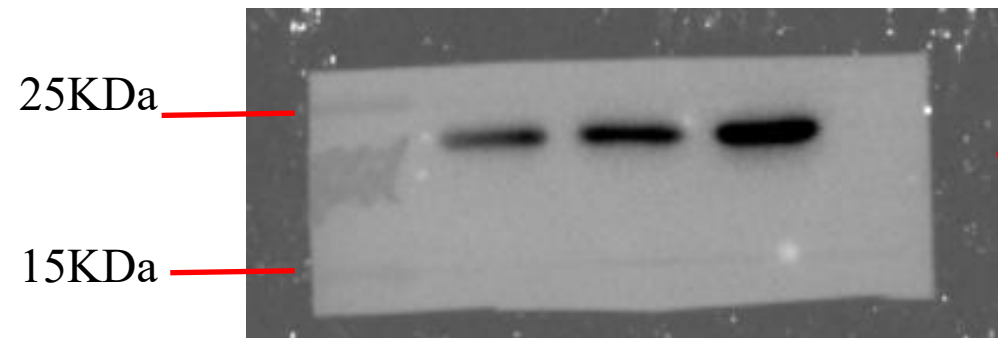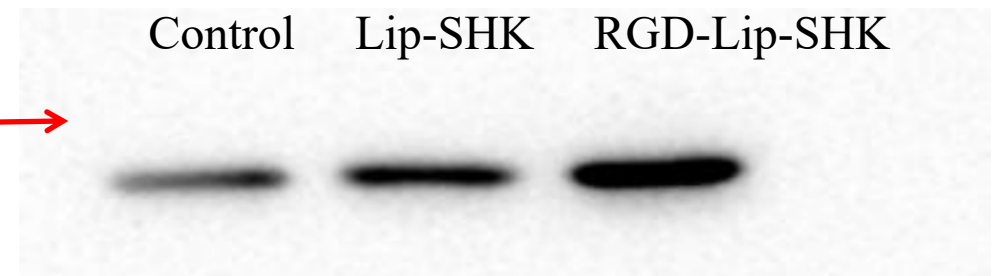

Bcl-2 26KDa

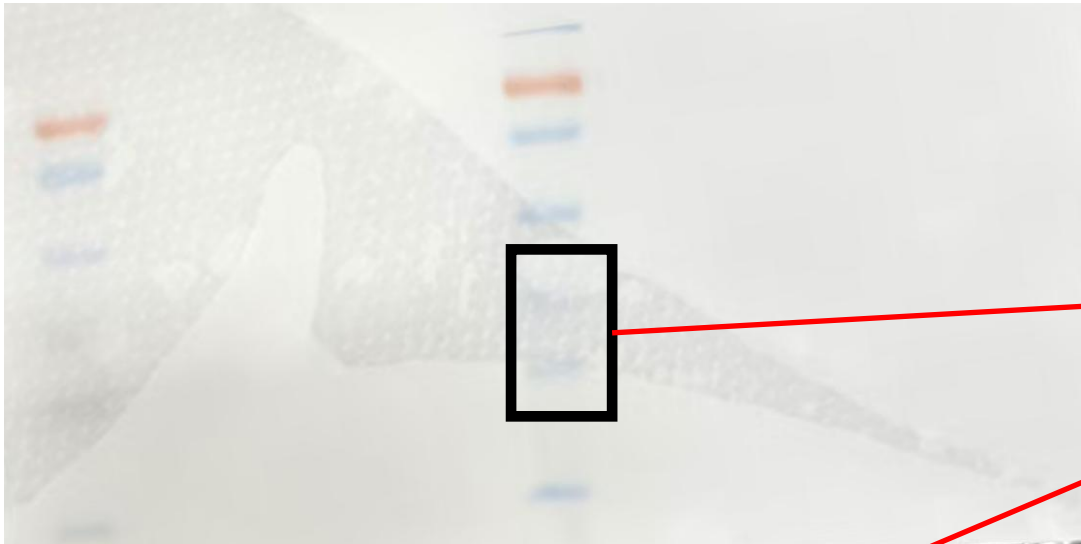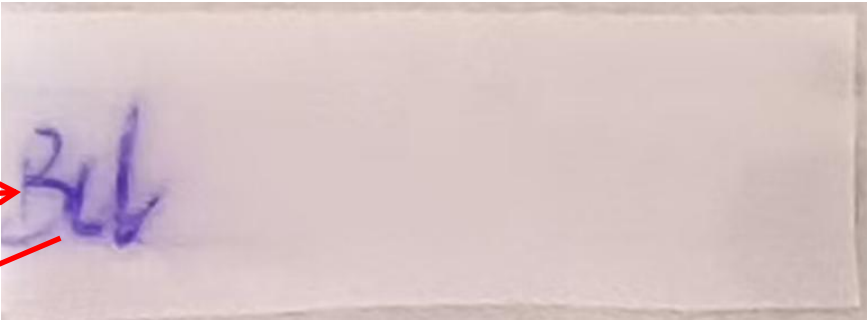

35KDa

25KDa

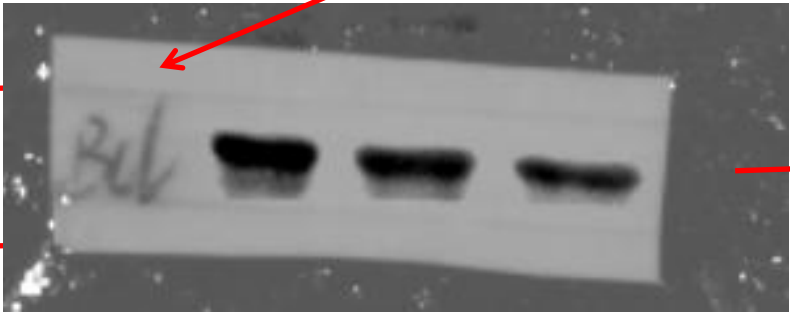

Control Lip-SHK RGD-Lip-SHK

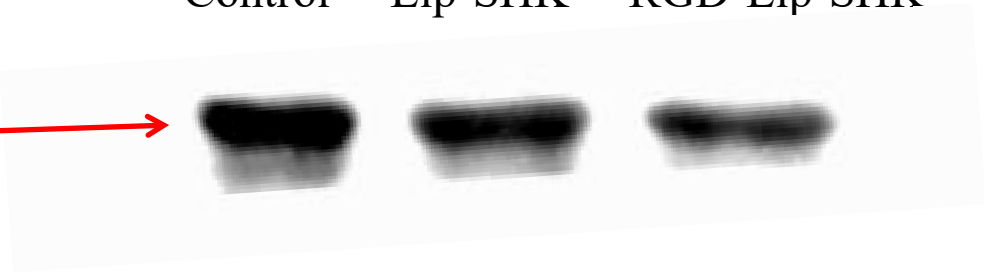

GAPDH 36KDa

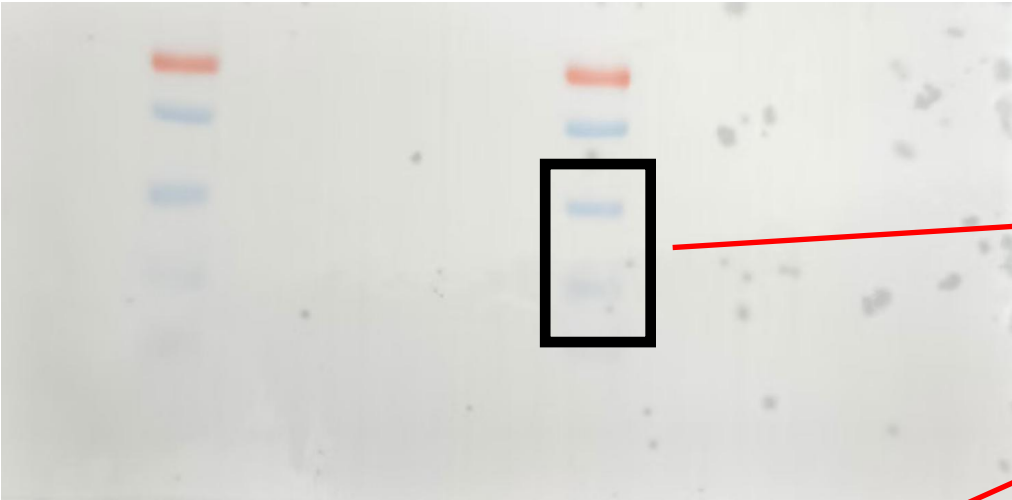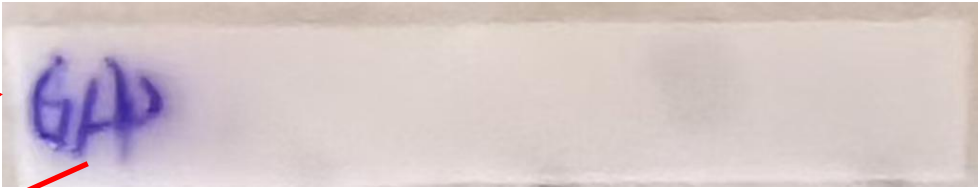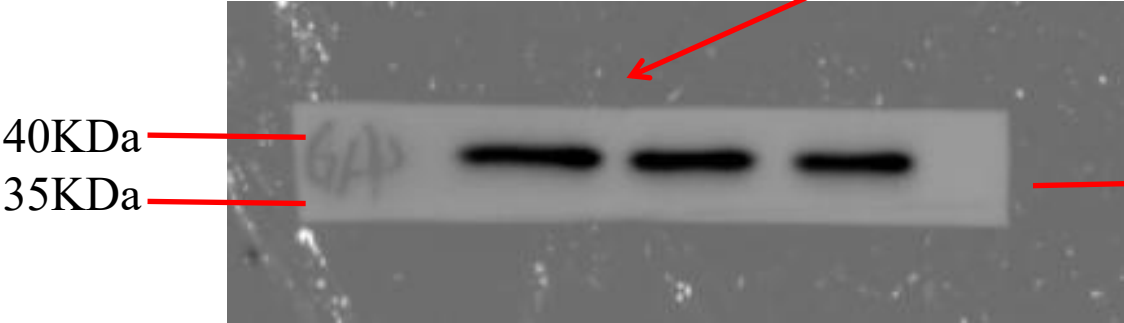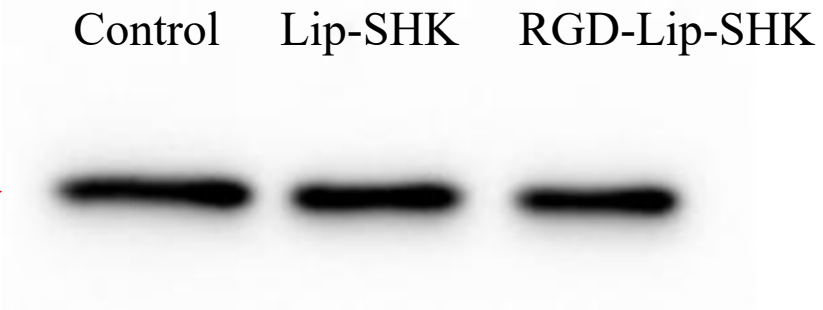

Supplement: Supplementary file 1 [file DataSheet1.pdf]
